# Posterior Cruciate Ligament Reconstruction Improves Sexual Health Postoperatively

**DOI:** 10.1016/j.asmr.2024.101041

**Published:** 2024-11-09

**Authors:** Riccardo D’Ambrosi, Federico Valli, Pietro Marchetti, Nicola Ursino, Amit Meena

**Affiliations:** a IRCCS Ospedale Galeazzi – Sant’Ambrogio, Milan, Italy; b Università degli Studi di Milano, Dipartimento di Scienze Biomediche per la Salute, Milan, Italy; c Università Tor Vergata, Roma, Italy (P.M.); and Shalby Hospital, Jaipur, India

## Abstract

**Purpose:**

To investigate changes in sexual function and quality of life following arthroscopic posterior cruciate ligament (PCL) reconstruction.

**Methods:**

Patients aged between 18 and 45 years who underwent PCL reconstruction were considered for inclusion. At the time of hospital admission, each patient was asked to complete the New Sexual Satisfaction Scale-Short Form, before injury (preinjury) and at the time of hospital admission (preoperative). In addition, patients were followed for a minimum of 24 months (final follow-up). Quality of life was measured using the Anterior Cruciate Ligament Quality of Life Questionnaire at hospital admission (preoperative) and at the 24-month follow-up visit (final follow-up).

**Results:**

A total of 35 patients were included in the study; 24 (68.6%) were male, and 11 were female (31.4%). The mean age at the time of surgery was 29.23 *±* 7.52 years, and the mean follow-up duration was 52.97 *±* 22.69 months. In all patients, the hamstring tendon was used. Sexual activity at the last follow-up visit was significantly different from that preoperatively (*P* = .024), while no difference was observed between the preinjury and last follow-up values (*P* = . 243). Sexual activity was more frequent before injury than preoperatively (*P* = . 009). Quality of life at the last follow-up visit was significantly better than that preoperatively (*P* < .001).

**Conclusions:**

Reconstructive surgery after PCL injury improves the sexual health of patients regardless of age or sex.

**Level of Evidence:**

Level III, retrospective cohort study.

Posterior cruciate ligament (PCL) tears are serious injuries that have substantial long-term consequences for the knee joint. Although solitary PCL tears are considered rare, with an estimated prevalence of 1% to 6%, structural damage to the PCL is found in up to 38% of trauma patients with acute knee injuries accompanied by hemarthrosis.^1^, ^2^, ^3^ Therefore, more than 60% of PCL injuries are linked to other ligament and capsule injuries. Among patients with PCL injuries, combined PCL and posterolateral corner injuries are most common, with a prevalence ranging from 15% to 42%.^4^ Because of a greater frequency of involvement in trauma, male patients are more susceptible to PCL injuries than are female patients, and PCL injuries typically occur between the ages of 28 and 34 years.^5^^,^^6^

Sexual activity is a fundamental aspect of human life.^7^^,^^8^ Its physical, emotional, psychological, and social components have a widespread influence on various facets of our lives. Sexual health and functioning impact quality of life (QoL). QoL declines when sexual dysfunction is present. In a nationally representative sample consisting of 3,515 sexually active U.S. adults, 62.2% of men and 42.8% of women considered sexual health to be “highly important” to QoL.^7^ Most research on the relationship between sex and QoL has focused on clinical populations with particular pathological diseases to investigate the impact of these conditions on patients' sexual well-being, but no studies have analyzed the correlations between PCL injuries and sexual activity.^7^

The anterior cruciate ligament (ACL) and PCL are essential for knee stability; after injuries, they can compromise knee stability and proprioceptive sensibility, resulting in instability, weakness, and diminished leg strength. Individuals may refrain from resuming their physical activity routines and may avoid exerting pressure on their knees. Furthermore, individuals may not recover the knee functions essential for sexual activity.^5^^,^^9^

The purpose of this study was to investigate changes in sexual function and QoL after arthroscopic PCL reconstruction. It was hypothesized that sexual function would improve after arthroscopic PCL reconstruction.

## Methods

### Study Protocol

In this study, all procedures involving human participants were performed in compliance with the 1964 Helsinki Declaration and its later amendments.^10^ The present study was approved by the Ethics Committee of the IRCCS, Ospedale San Raffaele (ACL-L2104). The study was conducted according to the Strengthening the Reporting of Observational Studies in Epidemiology checklist. Informed consent was obtained from all the participants.^11^

### Eligibility Criteria

Inclusion criteria were patients aged between 18 and 45 years, anatomical single-bundle arthroscopic PCL reconstruction, with a hamstring graft, the same rehabilitation protocol after surgery, followed for a minimum of 24 months, and less than 6 months from injury to surgery. Exclusion criteria were previous surgery (except arthroscopic meniscectomy); surgical-site infection; bilateral PCL injuries; additional injuries, such as anterior cruciate ligament, medial collateral ligament, lateral collateral ligament, or posterolateral corner injury; no sexual activity; and additional internal, urologic, or psychiatric problems that could affect sexual activity. Concomitant meniscal treatment (meniscal suture or meniscectomy) was not considered an exclusion criterion.

### Clinical Assessment

Clinical assessments were conducted by 2 independent clinicians who were not involved in the clinical management of patients. At the time of hospital admission, each patient was asked to complete the New Sexual Satisfaction Scale-Short Form (NSSS-S), which was composed of questions inquiring about their sexual activity before injury (preinjury) and at the time of hospital admission (preoperative). In addition, patients were followed for a minimum of 24 months (final follow-up). The NSSS-S is a 12-item instrument that measures global sexual satisfaction regardless of sex/gender, sexual orientation, or relationship status (Appendix Table 1, available at www.arthroscopyjournal.org). The theoretical framework for this scale is based on Bancroft, Loftus, and Long’s “three windows” approach, in which the first focuses on personal habits, perceptions, and feelings; the second window focuses on emotional exchange with partners; and the third window focuses on sexual activities. There are 2 subscales of the NSSS-S: the individual focus subscale and the interpersonal/activity focus subscale.^12^

**Table 1:** Sample Characteristics (N = 35)

	n (%)
Sex	
Female	11 (31.4)
Male	24 (68.6)
Age, yr	
<30	18 (51.4)
≥30	17 (48.6)
Side	
Right	15 (42.9)
Left	20 (57.1)
Graft	
Hamstring	35 (100.0)

	Mean ± Standard Deviation	Median [Interquartile Range]
Age	29.23 *±*7.52	28.00 [22.00, 36.00]
Follow-up length, mo	52.97 *±*22.69	42.00 [34.00, 80.00]

QoL was measured using the Anterior Cruciate Ligament Quality of Life Questionnaire (ACL-QoL) at hospital admission (preoperative) and at the 24-month follow-up visit (final follow-up). The Quality of Life Outcome Measure (Questionnaire) for ACL-QoL is an instrument developed with the objective of evaluating the QoL of patients with chronic ACL injury; it contains 31 items that are subdivided into 5 domains: Symptoms and Physical Complaints, Work-Related Concerns, Recreation Activities and Sport Participation or Competition, Lifestyle and Social and Emotional Aspects. Both instruments have been translated into several languages and adapted, and their measurement properties have been tested and have been shown to have good reliability and responsiveness^13^^,^^14^ (Appendix Table 2, available at www.arthroscopyjournal.org).

**Table 2:** Evaluation of Sexual Activity and Quality of Life

	Mean ± SD	Median [IQR]	Pairwise Comparisons (*P* Value)		
			Preinjury vs Preoperative	Preinjury vs Last Follow-Up	Preoperative vs Last Follow-Up
NSSS-S					
Preinjury	51.91 ± 6.02	53.00 [48.00, 56.50]	.009∗	.243	.024∗
Preoperative	48.43 ± 3.49	49.00 [46.50, 50.00]			
Last follow-up	51.31 ± 5.60	53.00 [48.00, 56.00]			
ACL-QOL					
Preoperative	31.94 ± 4.14	33.00 [29.00, 35.00]			<.001∗
Last follow-up	81.94 ± 3.69	82.00 [80.00, 84.00]			

NOTE. N = 35, Bonferroni adjustment was used for NSSS-S pairwise comparisons.

ACL-QOL, the Anterior Cruciate Ligament Quality of Life Questionnaire; IQR, interquartile range; NSSS-S, New Sexual Satisfaction Scale-Short Form; SD, standard deviation.

∗ Statistically significant value.

### Statistical Analysis

Descriptive data are expressed as the mean and standard deviation, median and interquartile range or absolute number and percentage frequency. The normality of the distribution of continuous variables was tested with the Shapiro-Wilk test. Paired *t* tests were performed to compare scores measured at different time points. Bonferroni adjustment was used for the NSSS-S score. The relationships between the NSSS-S and ACL-QoL scores were evaluated using Pearson correlations. All tests were 2 tailed. A *P* value less than .05 indicated statistical significance. All the statistical tests were performed with R, version 4.3.0 (R Foundation for Statistical Computing, Vienna, Austria; https://www.R-project.org/).

## Results

### Demographic Data

A total of 35 patients were included in the study, 24 (68.6%) were male and 11 were female (31.4%). The mean age at the time of surgery was 29.23 *±* 7.52 years (range 18-45 years), and the mean follow-up duration was 52.97 *±* 22.69 months (range 25-87 months). In all (100%) patients, the hamstring tendon was used. The details are reported in Table 1.

### Sexual Activity and QoL

Sexual activity at the last follow-up visit was significantly different from that preoperatively (*P* = .024), whereas no difference was found between that preinjury and that at the last follow-up visit (*P* = . 243). Sexual activity was more frequent preinjury than preoperatively (*P* = . 009). QoL at the last follow-up visit was significantly better than the preoperative QoL (*P* < .001). The detailed results are reported in Table 2.

### Correlations

The only significant correlation was found for NSSS-S between preinjury and final follow-up value (rho = 0.95; *P* = .001). The detailed results are reported in Table 3.

**Table 3:** Correlations Between ACL-QOL and NSSS-S

		Rho	*P* Value
NSSS-S			
Preinjury	Preoperative	0.16	.372
Preinjury	Last follow-up	0.95	<.001∗
Preoperative	Last follow-up	0.18	.302
ACL-QOL			
Preoperative	Last follow-up	0.15	.401
ACL-QOL	NSSS-S		
Preoperative	Preinjury	0.03	.888
Preoperative	Preoperative	−0.15	.380
Preoperative	Last follow-up	−0.02	.917
Last follow-up	Preinjury	−0.24	.175
Last follow-up	Preoperative	−0.11	.538
Last follow-up	Last follow-up	−0.25	.148

NOTE. N = 35.

ACL-QOL, the Anterior Cruciate Ligament Quality of Life Questionnaire; IQR, interquartile range; NSSS-S, New Sexual Satisfaction Scale-Short Form; SD, standard deviation.

∗ Statistically significant value.

## Discussion

The main findings of our study indicate that posterior cruciate ligament reconstruction (PCLR) can have a beneficial effect on sexual health, but because of the limitations of the study, these results should be interpreted with caution. Although there is evidence of abnormal tibiofemoral kinematics in people with PCL deficiency, no definitive prognostic indicators have been identified for determining which individuals may experience disability, discomfort, or osteoarthritis as the result of chronic PCL insufficiency. Moreover, inconsistencies between subjective and objective outcome assessments frequently occur in individuals with PCL injuries.^15^

Pain, in our opinion, is the root cause of reduced proprioceptive function, diminished extensor muscle strength, and reduced sexual concentration of sensations of insecurity. Sexual dysfunction is likely also influenced by posttraumatic psychological variables.

Activity of daily living consequences and reduced QoL, including sexual activity, can occur. This subjective sexual activity as a new endpoint, also in knee surgical areas, has become an important factor in the rating of treatment results

Nonsurgical therapy is often recommended for individuals with isolated grade I or II PCL injuries, as well as for those with grade III injuries exhibiting minor symptoms or modest activity demands. Patients must be advised that nonsurgical management is not curative and that symptoms such as discomfort, instability, and activity restrictions may persist or worsen despite adhering to the nonsurgical rehabilitation protocol.^15^

Operative treatment of PCL injuries is recommended for patients who have symptomatic grade III (complete) tearing and have not improved functionally in response to nonoperative treatment. In addition, patients who have PCL injuries that are accompanied by intra-articular or capsuloligamentous injuries, or who have high-grade knee laxity, should be evaluated for conservative treatment. A failure rate of 1% to 25% is reported after primary PCL reconstruction, which increases to 45% when subjective failure is defined as unfavorable patient-reported outcomes.^5^^,^^15^

The study suggested that when choosing between surgical or conservative treatment, it is important to inquire about the patient’s sexual activity and postoperative expectations. Flynn et al.^7^ recently showed that more than 50% of sexually active men and more than 40% of sexually active women of all ages consider sexual health highly important to their QoL. The results were consistent even among people who reported being in fair or poor health or having a chronic health condition. The scientists found a link between overall self-reported health and the perceived significance of sexual health. At least 70% of sexually active persons who assessed their general health as good felt sexual health to be highly important.^7^ Grinde^8^ also verified that the frequency of sexual engagement is closely linked to happiness.

Few orthopaedic studies have analyzed sexual activity after surgery. Mazlum et al.^16^ conducted a study to examine how ACL injury and its reconstruction affect men's sexual function. Similar to our findings, the authors demonstrated that ACL injury has a negative impact on sexual function. Sexual function after ACL surgery is contingent upon the surgical outcome. When determining the treatment for ACL damage, it is important to inquire about the patient’s sexual activity and their expectations after surgery.^16^

Sexual activity also has become a key topic in knee-replacement surgery. A study on sexual constraints in patients who underwent total knee arthroplasty was conducted.^17^ Before total knee arthroplasty, 45% of patients experienced restricted sexual quality and/or frequency because of their knee. Patients experienced an average of 17.1 months of sexual restriction before surgery, mostly as the result of discomfort (87%) and a reduced range of motion or flexibility (44%). Fifty-five percent of patients had to adjust their sexual posture because of their knee, and 97% of them specifically mentioned avoiding kneeling during sex. After surgery, a lower percentage of patients needed to change their sexual position because of their knee (55% vs 28%) and avoid putting weight on the affected knee during sexual activity (97% vs 79%). Patients returned to sexual activity within an average of 2.4 months, with a range from 0 to 18 months. Although there were overall enhancements, 25% of patients experienced reduced sexual activity in the first year after the procedure. After 1 year of recovery, 60% of the patients found it easier to engage in sexual activity compared with the previous year. Among these patients, 84% reported experiencing less pain, and 30% noted an increase in mobility or range of motion.^17^

Recently, Ihle et al.^18^ evaluated occupational consequences after isolated reconstruction in cases of chronic PCL insufficiency finding that operative treated patients with a chronic PCL insufficiency achieve an improvement of the clinical result. Patients with low physical load at their workplace achieve less restrictions.

Ochiai et al.^19^ evaluated the treatment outcome of PCLR using the Medical Outcome Study 36-item Short-Form Health Survey (SF-36), a patient-based QoL questionnaire comparing it with anterior cruciate ligament reconstruction (ACLR). In the ACL group, all evaluation methods showed significant improvement after surgery. In the PCL group, however, improvement was observed in only 3 of 8 subscales of the SF-36, Lysholm score, and posterior tibial translation after surgery. In intergroup comparison, the PCL group showed inferior performance in three subscales of the SF-36, Lysholm score and ROM for flexion compared with the ACL group.^19^

In 2023, Winkler et al.^20^ compared patient-reported outcomes after isolated ACLR, isolated PCLR, and combined ACLR and PCLR after at least 2 years of follow-up. The authors revealed clinically relevant improvements in knee function after isolated ACLR, isolated PCLR, and combined ACLR/PCLR. Functional improvements in the sport and recreation subscale of the Knee injury and Osteoarthritis Outcome Score were particularly pronounced, indicating the importance of knee stability for sports activity.

Similarly, Filbay et al.^21^ reported that patients evaluated with a knee-specific measure (the Knee injury and Osteoarthritis Outcome Score-QoL) were more inclined to report lower health-related QoL values than the normal patients were. Revision operations, meniscal injuries, and significant radiographic osteoarthritis were linked to worse QoL after ACLR.

We used the NSSS-S to evaluate sexual activity in our group of patients during the trial. Currently, there is no universally agreed-upon definition of sexual satisfaction, and the existing overlap between predictors and criteria makes it challenging to establish a definitive criterion or tool for evaluating sexual satisfaction. The NSSS-S is a condensed version of the New Sexual Satisfaction Scale. The New Sexual Satisfaction Scale was developed by considering sexual satisfaction from 3 perspectives: individual fulfillment, interaction with partners, and specific behaviors that enhance enjoyment. A recent psychometric investigation verified that the NSSS-S has robust psychometric support as a bidimensional gauge of sexual satisfaction.^12^

According to current literature and in line with our results, PCL injury causes worsening of sexual function scores in parallel with QoL and reconstructive of the ligament causes improvement in these scores. The success of reconstruction affects overall sexual satisfaction. According to the results of the current study, when deciding between surgical or conservative treatment, the patient's expectations for their sexual life after the operation should be considered.

### Limitations

The study includes only 35 participants, which is inadequate for making generalized conclusions about the broader population. Given the small sample size, the study lacks statistical power, making it difficult to detect differences that could be significant in a larger cohort. The retrospective design of the study, particularly concerning preinjury sexual activity, introduces substantial recall bias.

Furthermore, the 24-month follow-up may not be sufficient to capture the long-term outcomes of PCL reconstruction on sexual function and QoL. Issues such as the onset of degenerative changes, chronic pain, or the eventual impact of surgical complications may not fully manifest within this time frame, leading to an incomplete understanding of the true long-term effects.

Patients were asked to recall their sexual activity before the injury, which might be distorted by memory errors or the current impact of their condition. This approach undermines the reliability of the preinjury data, making it difficult to draw accurate comparisons between preinjury and postreconstruction sexual activity.

Sexual function is influenced by myriad factors, including psychological and emotional states, which were not adequately addressed in this study. By focusing purely on physical aspects of sexual activity, the study fails to account for the complex interplay of emotional well-being and mental health that could significantly affect sexual satisfaction and QoL. This omission weakens the comprehensiveness of the research findings. Finally, there's a selection bias; in fact, the patients included in the study have already decided to have surgery, presumably as the result of instability and/or poor function in other aspects than sexual health. The results are not going to be applicable to all patients with isolated PCL injuries.

## Conclusions

Reconstructive surgery after PCL injury improves the sexual health of patients regardless of age or sex.

## Disclosures

All authors (R.D., F.V., P.M., N.U., A.M.) declare that they have no known competing financial interests or personal relationships that could have appeared to influence the work reported in this paper.
