# Supplementary material for: Posterior Cruciate Ligament Reconstruction Improves Sexual Health Postoperatively
Source: Arthrosc Sports Med Rehabil. 2024 Nov 9;7(2):101041. doi: 10.1016/j.asmr.2024.101041 (PMC12034056; doi:10.1016/j.asmr.2024.101041)
Supplement: Supplementary Appendix 1 [file mmc1.pdf]

Appendix 1

The New Sexual Satisfaction Scale-Short Form (NSSS-S)

	**Not at all Satisfied**	**A Little Satisfied**	**Moderately Satisfied**	**Very Satisfied**	**Extremely Satisfied.**
The quality of my orgasms					
My "letting go" and surrender to sexual pleasure during sex					
The way I sexually react to my partner					
My body's sexual functioning					
My mood after sexual activity					
The pleasure I provide to my partner					
The balance between what I give and receive in sex					
My partner's emotional opening up during sex					
My partner's ability to orgasm					
My partner's sexual creativity					
The variety of my sexual activities					
The frequency of my sexual activity					

The Anterior Cruciate Ligament Quality Of Life Questionnaire

Anterior Cruciate Ligament Quality Of Life Questionnaire

Symptoms and Physical Complaints: **Circle the number that best describes your problem during the past month?**

1a. With respect to your overall knee function. How troubled are you by giving way episodes and what is the severity of your giving way episodes?

Major giving way 0 10 20 30 40 50 60 70 80 90 100 **Minor giving way**

1b. With respect to your overall knee function. What is the frequency of your giving way episodes?

Constantly giving way 0 10 20 30 40 50 60 70 80 90 100 **Never giving way**

2. With any kind of prolonged activity (i.e., greater than half an hour) how much pain or discomfort do you get in your knee?

Severe Pain 0 10 20 30 40 50 60 70 80 90 100 **No pain at all**

3. With respect to your overall knee function, how much are you troubled by stiffness or loss of motion in your knee?

Severely troubled 0 10 20 30 40 50 60 70 80 90 100 **Not troubled at all**

4. Consider the overall function of your knee and how it relates to the strength of your muscles. How weak is your knee?

Extremely weak 0 10 20 30 40 50 60 70 80 90 100 **Not weak at all**

Work-Related Concerns: **The following questions are being asked with respect to your job or vacation during the past month. Consider all the types of work together (full-time student, home maker, or any part time work).**

If your currently not employed for other reasons other than your knee, then place a check in this box.

5. How much trouble do you have, because of your knee, with turning or pivoting motions at work?

Severely troubled 0 10 20 30 40 50 60 70 80 90 100 **No trouble at all**

6. How much trouble do you have because of your knee with squatting motions at work?

Severely troubled 0 10 20 30 40 50 60 70 80 90 100 **No trouble at all**

7. How much of a concern is it for you to miss days from work due to your problems or re-injury to your knee?

Extremely concerned 0 10 20 30 40 50 60 70 80 90 100 **No concern at all**

8. How much of a concern is it for you to lose time from "school" or work because of the treatment of your ACL-deficient knee?

Extremely concerned 0 10 20 30 40 50 60 70 80 90 100 **No concern at all**

Recreational Activities And Sports Participation: **The following questions are concerned with your ability to function and participate in these activities as they relate to your ACL-deficient knee. Consider the last month.**

9. How much limitation do you have with sudden twisting and pivoting movements or changes in direction?

Totally limited 0 10 20 30 40 50 60 70 80 90 100 **No limitations**

10. How much of a concern is it for you that your sporting or recreational activities may result in the status of your knee worsening?

Extremely concerned 0 10 20 30 40 50 60 70 80 90 100 **No concern at all**

11. How does your current level of athletic or recreational performance compare with your pre-injury level?

Totally limited 0 10 20 30 40 50 60 70 80 90 100 **No limitations**

12. With respect to the activities/sports that you currently desire to be involved with, how much have your expectations changed because of the status of your knee?

Expectations totally lowered 0 10 20 30 40 50 60 70 80 90 100 **Expectations not lowered at all**

13. Do you have to play your recreational or sport under caution?

Always play under caution 0 10 20 30 40 50 60 70 80 90 100 **Never play under caution**

14. How fearful are you of your knee giving way when playing recreation or sport?

Extremely fearful 0 10 20 30 40 50 60 70 80 90 100 **No fear at all**

15. Are you concerned about environmental conditions such as a wet playing field, a hard court, or the type of gym floor when involved in your recreation or sport.

Extremely concerned 0 10 20 30 40 50 60 70 80 90 100 **Not concerned at all**

16. Do you find it frustrating to have to consider your knee with respect to your recreation or sport?

Extremely frustrated 0 10 20 30 40 50 60 70 80 90 100 **Not frustrated at all**

17. How difficult is it for you to "go full out" at your recreation or sport?

Extremely difficult 0 10 20 30 40 50 60 70 80 90 100 **Not difficult at all**

18. Are you fearful of playing contact sports? If you do not play contact sports for reasons other than your knee, check this box.

Extremely fearful 0 10 20 30 40 50 60 70 80 90 100 **No fear at all**

19. How limited are you in playing your most important sport or recreational activity?

Extremely limited 0 10 20 30 40 50 60 70 80 90 100 **Not limited at all**

20. How limited are you in playing your second most important sport or recreational activity?

Extremely limited 0 10 20 30 40 50 60 70 80 90 100 **Not limited at all**

<u>Life Style:</u> **The following questions are concerned with your life style in general and should be considered outside of your work and recreational or sport activities as they relate to your ACL deficient knee. Consider the last month.**

21. Do you have to concern yourself with general safety issues (i.e., carrying small children, working in the yard) with respect to your ACL-deficient knee?

Extremely concerned 0 10 20 30 40 50 60 70 80 90 100 **No concern at all**

22. How much has your ability to exercise and maintain fitness been limited by your knee problem?

Totally limited 0 10 20 30 40 50 60 70 80 90 100 **Not limited at all**

23. How much has your enjoyment of life been limited by your knee problem?

Totally limited 0 10 20 30 40 50 60 70 80 90 100 **Not limited at all**

24. How often are you aware of your knee problem?

All the time 0 10 20 30 40 50 60 70 80 90 100 **None of the time**

25. Are you concerned about your knee with respect to life style activities that you and your family do together?

Extremely concerned 0 10 20 30 40 50 60 70 80 90 100 **No concern at all**

26. Have you modified your life style to avoid potentially damaging activities to your knee?

Totally modified 0 10 20 30 40 50 60 70 80 90 100 **No modifications**

<u>Social And Emotional:</u> **The following questions are about your attitudes and feelings as they relate to your ACL-deficient knee. Consider the last month.**

27. Does it concern you that your competitive needs are no longer being met because of your knee problem?

Extremely concerned 0 10 20 30 40 50 60 70 80 90 100 **Not concerned at all**

28. Have you had difficulty being able to psychologically "come to grips" with your knee problem?

Extremely difficult 0 10 20 30 40 50 60 70 80 90 100 **Not difficult at all**

29. How often are you apprehensive about your knee?

All the time 0 10 20 30 40 50 60 70 80 90 100 **None of the time**

30. How much are you troubled with lack of confidence in your knee?

Severely troubled 0 10 20 30 40 50 60 70 80 90 100 **No trouble at all**

31. How fearful are you of re-injuring your knee?

Extremely fearful 0 10 20 30 40 50 60 70 80 90 100 **No fear at all**
